# Sequential automatic on-line determination of aquiculture nutrients: phosphate and nitrate

**DOI:** 10.1155/S1463924692000324

**Published:** 1992

**Authors:** P. Linares, M. D. Luque de Castro, M. Valcárcel

**Affiliations:** Department of Analytical Chemistry Faculty of Sciences University of Córdoba Córdoba E-14004 Spain

## Abstract

A continuous-flow method for the determination of nitrate and phosphate in water is proposed. The method is based on insertion of reagents and sample solutions into a single channel through a programmable switching valve. The method depends upon heteropolyacid with phosphate and on the modified Griess reaction for nitrate, and permits the determination of PO^-3^_4_ in the range 1-20 μg/ml and NO^-^_3_ between 5 and 100 μg/ml, the achievable sampling frequency being 45/hour. The two analytes can be determined in N/P ratios between 0.25 and 100. The method has been applied to the determination of nitrate and phosphate in the sea-water used in fish farms.


Journal of Automatic Chemistry, Vol. 14, No. 5 (September–October 1992), pp. 173–175

### Sequential automatic on-line determination of aquiculture nutrients: phosphate and nitrate

**P. Linares, M. D. Luque de Castro and M. Valcárcel**

*Department of Analytical Chemistry, Faculty of Sciences, University of Córdoba, E-14004 Córdoba, Spain*

*A continuous-flow method for the determination of nitrate and phosphate in water is proposed. The method is based on insertion of reagents and sample solutions into a single channel through a programmable switching valve. The method depends upon heteropolyacid with phosphate and on the modified Griess reaction for nitrate, and permits the determination of $PO_4^{3-}$ in the range 1–20 μg/ml and $NO_3^-$ between 5 and 100 μg/ml, the achievable sampling frequency being 45/hour. The two analytes can be determined in N/P ratios between 0·25 and 100. The method has been applied to the determination of nitrate and phosphate in the sea-water used in fish farms.*

On-line monitoring and multideterminations are primary goals for analytical chemists as the demand for this type of measurement in the industrial and biotechnological fields is growing. Unsegmented-flow techniques lend themselves to this goal, as demonstrated by the large number of applications reported in the last few years [1], most of which relied on either of the two major unsegmented-flow approaches – completely continuous-flow analysis [2] and flow-injection analysis [3,4]. Excellent results have been achieved with methods for the simultaneous or sequential determination of silicate/phosphate [5], monochloramine/dichloramine/free chlorine [6], and nitrate/nitrite/ammonium [7]. The trend towards unsegmented flow techniques is likely to spread even further as a result of the large number of industry-university co-operative research centres established in the USA [8,9], which are slowly being imitated by other countries [10].

The aim of this work was to develop a new technique for on-line multideterminations; an unusual unsegmented-flow single-channel manifold was used, the key component of which is a programmable switching valve which allows the sample and the reagents to be selected sequentially. This approach was used to develop methods for the determination of phosphate and nitrate in natural waters for subsequent application in aquiculture, where monitoring (and control, if required) of these parameters is extremely important. The determination of nitrate is based on its reduction to nitrite by passage through a solid redox minicolumn packed with copperized cadmium and located in the sample stream. This is followed by the Griess diazotization reaction [11,12]. The determination of phosphate relies on the formation of its heteropoly acid with $MoO_4^{2-}$ and photometric detection of the Mo(V) as molybdenum blue obtained with a suitable reductant (for example ascorbic acid [13,14]).

### Experimental

### Reagents

The stock solutions used included a buffer containing 100 g/l $NH_4Cl$, 20 g/l $Na_2B_4O_7$ and 1 g/l EDTA; aqueous solutions of 5% sodium molybdate in 2 M $HNO_3$; 2% ascorbic acid containing 10% glycerin; 0·380 g of sulphanilamide dissolved in 4 ml of concentrated HCl and diluted to 100 ml with distilled water; and 0·140 g of N-(1-naphthyl)ethylenediamine and 1·600 g of NaCl dissolved in 100 ml with distilled water. Aqueous standard solutions of phosphate and nitrate were prepared from $KH_2PO_4$ and $KNO_3$ (from Merck). The reduction column was made by packing a glass capillary (10 cm long, 3 mm bore) with cadmium granules that were coated with copper by passing 100 ml of a solution of 0·1% cupric sulphate and 0·1 M of Cu-EDTA complex through the column at 1·0 ml/min for 2 h.

### Instruments and apparatus

A Unicam 8625 UV-Vis spectrometer equipped with a Hellma 178·12QS flow-cell (inner volume 18 μl) and connected to a Radiometer pHM 62 pH-meter; a four-channel Eppendorf Eva-peristaltic pump; and a nine-way 2500 Eppendorf Eva-switching valve were used. A personal computer (PC) equipped with a PC-ADDA/14 analogue-to-digital interface with 12 bit resolution for the data acquisition, a dual 'serial-control' interface for the pump and valve multi input-output, a 40 MB hard disk and a 3½ in floppy disk drive, and a STAR LC10 printer was also used. Tecator TMII chemifolds were also employed.

### FIA manifold

Figure 1 shows the manifold used, the central element of which is the programmable switching valve for insertion into the main channel (channel A) of the sample-reagents sequence required for derivatization prior to detection of the analytes. The nature of the stream circulating along channel A depends on the position of the switching valve (SV), between 1 and 4, in order to establish the previously programmed reagent-sample sequence. By sequentially selecting channel 1 (sample) and 3 (molybdate/ascorbic acid solution) phosphate can be determined. As the sample is aspirated through channel 2, nitrate is reduced to nitrite in the solid reactor CR. The sequential change of the sample (channel 2) and reagents (sulphanilamide/N-(1-naphthyl)ethylenediamine) (channel 4) enables the determination of nitrate.

0142-0453/92 $3.00 © 1992 Taylor & Francis Ltd.

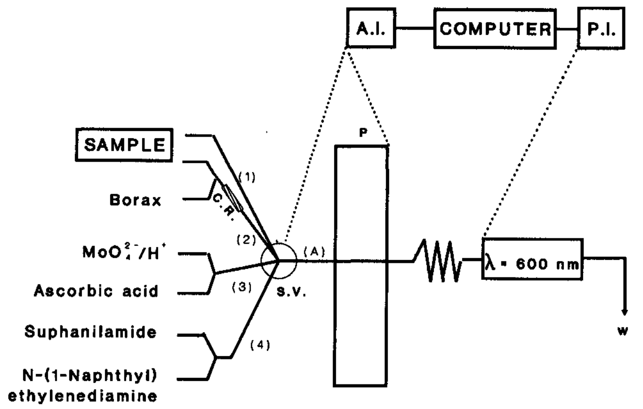

*Figure 1. Automatic unsegmented-flow manifold for the determination of phosphate and nitrate in water by sequential selection of sample and reagents. A.I. and P.I. denote active and passive interfaces, respectively; S.V. denotes switching valve, [A] main channel, P peristaltic pump and W waste (for details see text).*

The PC acquires the absorbance signals through the passive interface and converts them into phosphate and nitrate concentrations.

*Software*

The program controlling the manifold and performing data acquisition and processing was written in BASIC. The program can select the intervals for switching of the selecting valve to each position; and control the speed of the peristaltic pump, the intervals for data acquisition, the number of analyses to be carried out, the interval between consecutive analyses and the display of the maximum absorbance of the peak recorded on each sample insertion. Samples were inserted in triplicate and the average absorbance was calculated by comparison with the stored calibration graph. The results are displayed on screen and can be stored, printed, erased or even altered by rejecting some value.

### Results and discussion

Each method was optimized individually and a compromise configuration was adopted for the sequential determination of both analytes. The features of the chemical and flow systems and the values of the optimized variables are shown in table 1. The reagents used were those commonly employed for the determination of these analytes in waters (formation of the heteropoly acid for phosphate and the Griess reaction as modified by Shinn for nitrate). The selected physical variables and wavelength (600 nm) were a compromise between the optimal values for each determination.

*Table 1. Optimized values of the chemical and continuous systems*
*(a) Chemical systems.*

| Parameters Analytes | Reagents | Reaction medium |
|---|---|---|
| $PO_4^{3-}$ | 5% molybdate<br>2% ascorbic acid | 2M $HNO_3$<br>10% glycerin |
| $NO_3^-$ | 0·38% sulphanilamide<br>0·144% N-(1-naphthyl)-ethylenediamine | 4% HCl<br>1·6% NaCl |

*(b) Continuous flow system.*

| Parameters Analytes | q[a] (ml/min) | L[b] (cm) | λ (nm) |
|---|---|---|---|
| $PO_4^{3-}$ | 2·0 | 300 | 540 |
| $NO_3^-$ | 0·8 | 200 | 820 |
| Joint determination | 1·0 | 300 | 600 |

Where [a] = flow-rate and [b] L = reactor length.

Automatic control of the switching valve allowed the intervals for each position to be programmed, so the volume of sample and reagent could be changed as required, which, in turn, allowed the sensitivity of the method to be changed from 0·313 AU (absorbance units) to 0·609 AU for a concentration of 5μg/ml of phosphate, and from 0·144 AU to 0·586 AU for 50 μg/ml of nitrate when sample/reagent volumes were changed from 2000/40 to 2000/200 μl. The ratio in which phosphate and nitrate can be determined can also be changed in this way. Thus, the ratios can be altered between 4 : 1 to 1 : 100 phosphate and adjusted to the usual ratio of these analytes in the water used at a given fish farm.

The transient signals provided by the photometer are independent of the saline concentration in the sample, thus allowing the determination of these analytes in sea-water. The passivating effect of the high saline concentration on the redox reactor called for a longer reactor (10 cm long, 3 mm bore) than for tap-water.

### Features of the determination

The features of the sequential determination of these analytes are summarized in table 2. The sensitivity was acceptable in both cases, and the determination ranges encompass the concentrations required for monitoring these analytes in fish hatcheries and small tanks. The

*Table 2. Features of the methods.*

| Parameter | Equation | Regression coefficient | Linear range (μg/ml) | R.S.D. (%) |
|---|---|---|---|---|
| $PO_4^{3-}$ | $A = 0{\cdot}1082 + 3{\cdot}25\ 10^{-2}\ |PO_4^{3-}|$ | 0·9947 | 1·0–20·0 | ±2·0 |
| $NO_3^-$ | $A = 0{\cdot}0897 + 8{\cdot}08\ 10^{-3}\ |NO_3^-|$ | 0·9988 | 5·0–100·0 | ±3·0 |

precision was also acceptable for these determinations. The analytes can be determined by triplicated insertion of each sample, at a rate of 45 samples per hour.

### Analytical applications

The proposed method was applied to the determination of phosphate and nitrate in sea-water from a feeding stream for small tanks at a fish-breeding farm. The input and output of these tanks was continuously monitored in a laboratory experiment during 30 min intervals over a fortnight. The results obtained were compared with those provided by the batch methods, based on the same derivatizing reactions, by analysing the samples once daily. The results provided for both analytes by the proposed methods were mutually consistent and with those of the batch methods, thereby showing the accuracy of the proposed methods and the stability of these analytes in the samples (the concentrations remained constant over the fortnight at $7{\cdot}2 \pm 0{\cdot}2$ and $9{\cdot}6 \pm 0{\cdot}3$ for nitrate and $9{\cdot}4 \pm 0{\cdot}2$ and $6{\cdot}3 \pm 0{\cdot}1$ for phosphate for the inlet and outlet samples, respectively, expressed in μg/ml).

### Conclusions

The proposed methods allow phosphate and nitrate in sea-water samples to be monitored with a high degree of automation, and provides a good way for the routine daily determination of these parameters in fish farms. The ease and rapidity with which the results can be obtained allows immediate corrective actions to be taken, which is important in fish farming.

### Acknowledgement

The authors wish to thank the Comisión Interministerial de Ciencia y Tecnología (CICyT) for its financial support (under Grant No. MAR88-0112).

### References

1. Luque de Castro, M. D., *Talanta,* **36** (1989), 591–599.
2. Goto, M., *Trends in Analytical Chemistry,* **2** (1983), 92–101.
3. Valcárcel, M. and Luque de Castro, M. D., *Flow Injection Analysis: Principles and Applications* (Ellis Horwood, Chichester, 1987).
4. Ruzicka, J. and Hansen, E. H., *Flow Injection Analysis* (Wiley and Sons, New York, 1988).
5. Linares, P., Luque de Castro, M. D. and Valcárcel, M., *Talanta,* **33** (1986), 889–893.
6. Aoki, T., *Environmental Science and Technology,* **23** (1989), 46–50.
7. Malcolme-Lawes, D. J. and Pasquini, C., *Journal of Automatic Chemistry,* **10** (1988), 192–197.
8. Illman, D. L., *Trends in Analytical Chemistry,* **7** (1986), 164–172.
9. Callis, J. B., Illman, D. L. and Kowalski, B. R., *Analytical Chemistry,* **59** (1987), 624A-637A.
10. van der Linden, W. E., de Niet, G. and Bos, M., *Analytica Chimica Acta,* **216** (1989), 307–319.
11. Saltzmann, B. E., *Analytical Chemistry,* **26** (1954), 1949–54.
12. American Public Health Association and American Water Works Association, Standard Methods for the Examination of Water and Waste Water (14th edn, American Public Health Association, Washington, D.C., 1975, pp. 434).
13. Florence, T. M., *Talanta,* **29** (1982), 345–352.
14. Instituto de Acuicultura, Torre de la Sal, Castellón (private communication).

